# Study of mixed mode fracture toughness and fracture trajectories in gypsum interlayers in corrosive environment

**DOI:** 10.1098/rsos.171374

**Published:** 2018-01-24

**Authors:** Bao Xiankai, Tao Meng, Zhao Jinchang

**Affiliations:** 1 College of Mining Engineering, Taiyuan University of Technology, Taiyuan, Shanxi, China; 2 School of Civil Engineering, Inner Mongolia University of Science and Technology, Baotou, Inner Mongolia, China; 3 College of Chemical and Biological Engineering, Taiyuan University of Science and Technology, Taiyuan, Shanxi, China

**Keywords:** mixed mode fracture toughness, fracture trajectory, gypsum interlayers, hot brine, salt cavern

## Abstract

Based on the engineering background of water dissolving mining for hydrocarbon storage in multi-laminated salt stratum, the mixed mode fracture toughness and fracture trajectory of gypsum interlayers soaked in half-saturated brine at various temperatures (20°C, 50°C and 80°C) were studied by using CSNBD (centrally straight-notched Brazilian disc) specimens with required inclination angles (0°, 7°, 15°, 22°, 30°, 45°, 60°, 75°, 90°) and SEM (scanning electron microscopy). The results showed: (i) The fracture load of gypsum specimens first decreased then increased with increasing inclination angle, due to the effect of friction coefficient. When soaked in brine, the fracture toughness of gypsum specimens gradually decreased with increasing brine temperature. (ii) When soaked in brine, the crystal boundaries of gypsum separated and became clearer, and the boundaries became more open between the crystals with increasing brine temperature. Besides, tensile micro-cracks appeared on the gypsum crystals when soaked in 50°C brine, and the intensity of tensile cracks became more severe when soaking in 80°C brine. (iii) The experimental fracture envelopes derived from the conventional fracture criteria and lay outside these conventional criteria. The experimental fracture envelopes were dependent on the brine temperature and gradually expanded outward as brine temperature increases. (iv) The size of FPZ (fracture process zone) was greatly dependent on the damage degree of materials and gradually increased with increase of brine temperature. The study has important implication for the control of shape and size of salt cavern.

## 1. Introduction

Fracture toughness, which represents the ability to resist crack growth, is a critical mechanical parameter which has been widely applied in geotechnical engineering related fields including: structure reliability analysis (coal mining, tunnel, geothermal reservoirs, oil and gas storage underground), hydraulic fracturing, rock burst prevention and treatment and earthquake prediction. It is also an inherent attribute of a material, independent of shape and loading conditions of the specimen. Most studies on the fracture toughness of rocks were focused on mode I because the opening mode failure is frequently encountered [1–6]. However, due to the randomly distributed cracks, faults, joints and natural weak plane, actual cracks in rock blocks are often subjected to combined loading, and occur not only in tension but also in shear. When being subjected to combined mode load, the fractures may propagate along curvilinear paths rather than the direction of original crack. In practical engineering application, it is necessary to determine the direction of fracture initiation from per-cracks when an estimate of crack arrest is required. Consequently, in the field of geomechanics and geotechnical engineering, the study of fracture toughness and fracture trajectory (i.e. crack propagation paths) under mixed mode loading are an interesting research subject for scholars.

**Figure 1.**
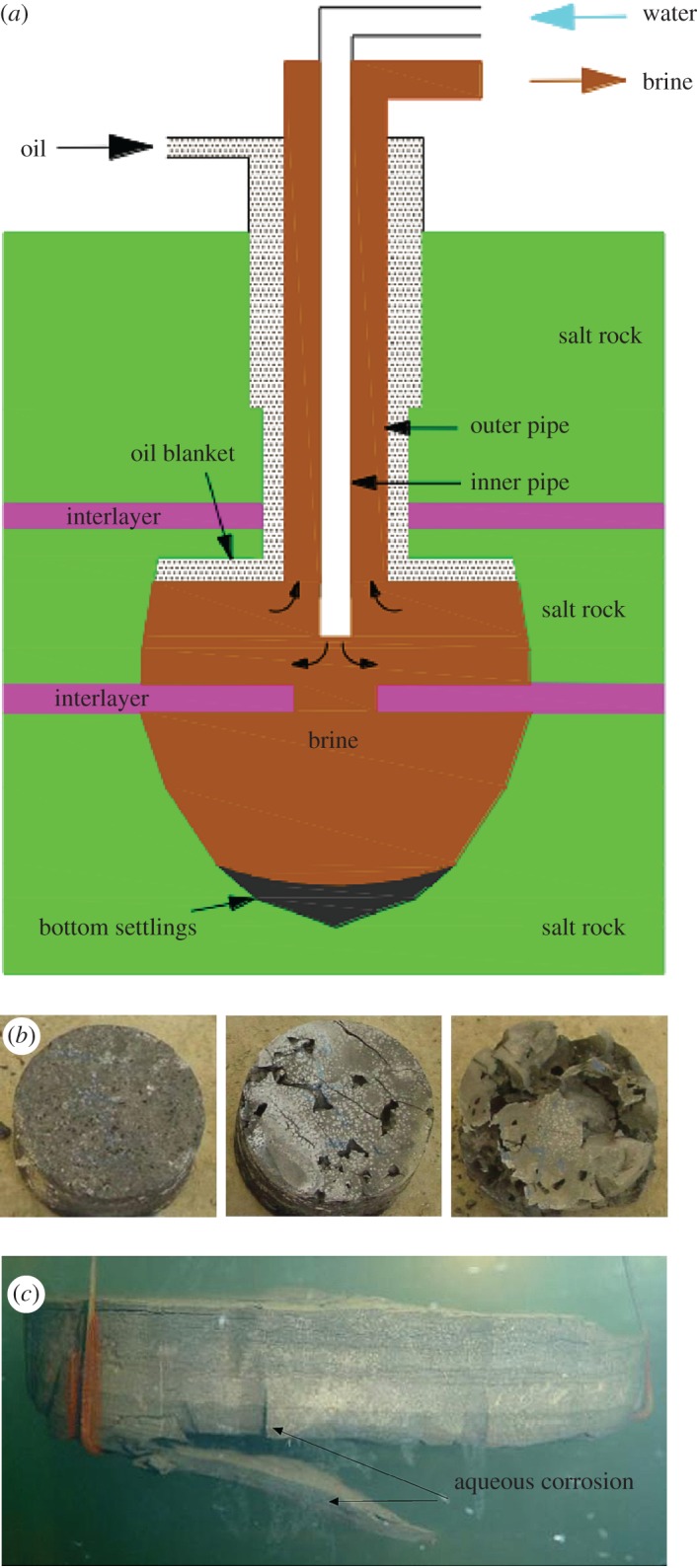
Corrosion process and collapse of insoluble interlayer in the process of solution mining of salt cavern [13].

Note that, in most cases, rock is always found to be saturated in water, brine or other corrosive liquid [7–12]. These corrosive liquids have been proven to have a significant impact on the property of rock (figure 1). Taking salt rock interlayers as an example, when forming salt caverns, rock salt around the cavity is dissolved in water or unsaturated brine, which results in suspended rock interlayers soaked in hot brine. The concentration of the brine varies with spatial position due to the effects of dissolution and gravity circulation. Specifically, the brine concentration is higher at the bottom and lower at the top of the salt cavern [14]. Hence, the rock interlayers are exposed to saline brines with different concentrations and different temperatures, and these brines damage and weaken the rock interlayers. The insoluble rock interlayers influence the flow field and impact the flow velocity. This has a pronounced impact on the rate of rock salt dissolution and makes it hard to control the shape and size of the salt cavern. Besides, due to the corrosion of hot brine and the huge volume of a salt cavern, rock interlayers will periodically collapse with increases in cavern size (figure 1). A sudden collapse of a thick interlayer may smash or cut the pipe. Damaged leaching pipes would change the depth of the water intake and the brine outlet, which causes an abnormal cavern shape [13]. It is worth noting that the phenomenon (the collapse of rock interlayers) depends on the fracture toughness and propagation trace of gypsum interlayers soaked in hot brine. However, the mixed mode fracture toughness and the propagation trace of rock interlayers under coupled thermo-hydro-chemical processes are not well understood, and few relevant data are available. Therefore, to accurately predict the caving pace of a hanging interlayer and to obtain the optimum size and shape of a salt cavern, it is necessary to investigate systematically the mixed mode fracture toughness and propagation trace of rock interlayers under different brine/temperature conditions.

The study of fracture toughness is also very necessary in the field of CO_2_ storage in saline aquifers [15]. Usually, the supercritical CO_2_ is injected into deep stratum. Owing to the difference in density of the supercritical CO_2_ and saline water, the supercritical CO_2_ is continuously moved upwards under the action of buoyancy. Eventually, the supercritical CO_2_ will encounter the dense gypsum caprock and be blocked by it. However, excessive CO_2_ will cause a larger pressure on the caprock. And, the increase in pressure inevitably acts upon the tightness of the gypsum caprock. When a critical internal pressure is reached, fracturing in the caprock may occur. That is, when the value of the stress intensity factor becomes greater than that of a certain critical stress intensity value of caprock, known as the fracture toughness, Kc, a fracture is presumed to be initiated. Note that, such fracturing is very possible because the fracture toughness of the caprock is relatively low. Therefore, in order to prevent the leakage of CO_2_, the study of mixed mode fracture toughness of gypsum caprock is very important.

Previously, several researchers have concentrated on the study of the mixed mode fracture toughness of rock, stress corrosion and salt interlayers. With regard to the research of mixed mode fracture toughness and stress corrosion, Aliha *et al.* [16] carried on the study of mixed mode fracture toughness using Schottky barrier diode (SBD) and cracked chevron notched Brazilian disc (CCNBD) specimens. They developed a new fracture criterion to predict the fracture initiation angle of rock under pure mode I, pure mode II and mixed mode I-II loading. Al-Shayea [17] studied the effect of confining pressures and temperature on fracture behaviour of rock. It was reported that the surrounding environment has a significant impact on the mechanical characteristic and fracture peak. Kataoka *et al.* [18,19] focused on the effect of water vapour on the fracture behaviour and crack propagation trace of granite. They concluded that the decrease of fracture toughness is contributed to the stress corrosion. Nasseri *et al.* [20] carried out a study to investigate the correlation between fracture toughness and fracture roughness using the specimens after heat treatment. The results showed that the fracture toughness decreases with the increase of temperature due to thermal cracking. With regard to the research of salt interlayers, Shi *et al.* [21] studied the brine concentration on the property of mudstone interlayers. It is reported that the weakening of mudstone interlayers will be more severe at higher brine concentrations. However, Jiang *et al.* [22] asserted that the effect of brine concentration can be neglected. Yu *et al.* [23] studied the concentration effect of hot brine on mechanical property of Xishan gypsum rock. They concluded that the mechanical properties of gypsum can be greatly affected by the brine concentration.

In summary, it can be considered that surrounding environment has a significant influence on the mechanical characteristics (e.g. microstructure, macrostructure) of rock. The resulting changes in microstructure, such as pores and fissures, will lead to changes in the macroscopic properties of the rock, including significant changes in fracture toughness and fracture trajectories [24–27]. However, none of the studies mentioned above address the fracture toughness and fracture trajectories of gypsum interlayers in corrosive environment (i.e. hot brine). Besides, minerals variably respond in the presence of chemical liquid depending on their crystal structure and chemical composition [28]. However, few relevant date about gypsum interlayers are available. Therefore, it is necessary to study the gypsum interlayers' fracture toughness and fracture trajectories under particular conditions. In this study, we conducted a serious of tests, including centrally straight-notched Brazilian disc (CSNBD) tests and scanning electron microscopy (SEM) tests to investigate the mixed mode fracture toughness and fracture trajectories of gypsum interlayers in corrosive environment. The conclusions would provide a certain theoretical guidance for the formation of salt caverns in China and also for evaluating their stability and integrity.

### 1.1. Experimental set-up and samples

**Figure 2.**
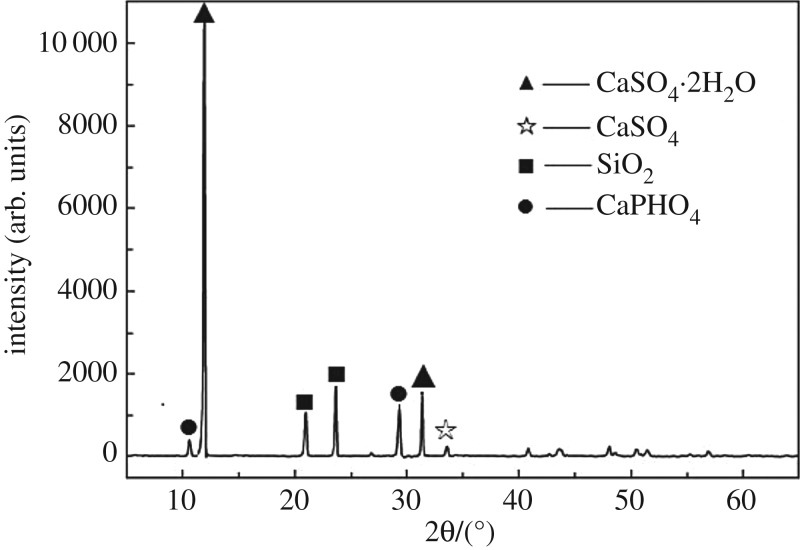
Analysis on X-ray diffraction of gypsum rock [29].

Gypsum interlayer cores were obtained from a bedded salt deposit, the Yunying salt formation. The gypsum (CaSO_4_⋅2H_2_O) content of this material is 85.4%. The composition and content of gypsum interlayers have been analysed by X-ray diffraction (figure 2 and table 1). The depth of most salt caverns is 500–2000 m, and the formation temperature is 20–80°C. As such, 20°C, 50°C and 80°C are used as the laboratory temperatures. In the process of constructing a salt cavern, the concentration of brine changes with changes in spatial position because of dissolution, the effect of gravity, and convective circulation effects. The brine concentration is higher at the bottom and lower at the top of the salt cavern. Specifically, the gypsum interlayers are usually located in the middle of salt cavern. Hence, in this paper, the half-saturated brine with various temperatures is used as the laboratory solution. All specimens were tested on a rock mechanics servo-controlled machine with a controlled displacement rate of 10^−3^ mm s^−1^. The precision of the deformation measurements recorded using this system can be as small as 10^−3^ mm [14].

**Table 1.** Compositions and contents of the tested specimens (mean value) [29].

chemical component	gypsum (CaSO_4_·2H_2_O)	quartz (SiO_2_)	Brushite (CaPHO_4_)	anhydrite (CaSO_4_)
content	85.4%	8.7%	4.8%	1.1%

**Figure 3.**
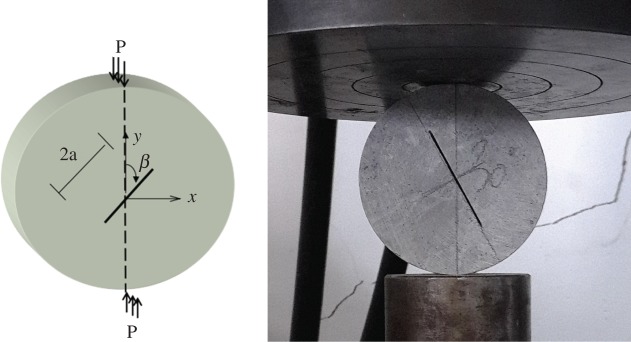
Schematic view of CSNBD specimen.

In this paper, centrally straight-notched Brazilian disc (CSNBD) specimens were used to determine the pure mode I, pure mode II and combined mode fracture strength (or fracture toughness) of gypsum (figure 3). The centre crack with required inclination angle was introduced by a double-side hacksaw with a 0.3 mm thick cutting edge. The geometrical features of the CSNBD specimen were as follows: radius *R* = 37.5 mm; the half crack length *a* = 18.75 mm; thickness *B* *=* 20 mm; *β* = 0°, 7°,15°, 22°, 30°, 45°, 60°, 75°, 90°, respectively.

The following mathematical formulae, put forward by Atkinson, were recommended to calculate the fracture toughness [30].

1.1 $$K_{I}=\frac{P\sqrt{a}}{\sqrt{\pi }RB}N_{I}$$

and

1.2 $$K_{II}=\frac{P\sqrt{a}}{\sqrt{\pi }RB}N_{II},$$

where $K_{I}$ is the mode I stress intensity factor; $K_{II}$ is the mode II stress intensity factor; *R* is the radius of the Brazilian disc; *B* is the thickness of the disc; *P* is the compressive load at failure (or fracture peak); *a* is the half crack length; and $N_{I}$, $N_{II}$ are non-dimensional coefficients which depend on *a/R* and the flaw inclination angle (*β*).

Atkinson *et al.* [30] developed $N_{I}$ and $N_{II}$ solutions for the CSNBD specimen given by five-term approximation (for *a/R* = 0.5).

1.3 $$N_{I}=\sum_{i=1}^{n}T_{i}{\left(\frac{a}{R}\right)}^{2i-2}A_{i}(\beta )$$

and

1.4 $$N_{II}=2\sin2\theta \sum_{i=1}^{n}S_{i}{\left(\frac{a}{R}\right)}^{2i-2}B_{i}(\beta ),$$

where the first five values of *T_i_* and *S_i_* and the corresponding $A_{i}(\beta )$ and $B_{i}(\beta )$ are given in tables 2 and 3, respectively.

**Table 2.** The first five values of *T_i_* and *S_i._*

	*T*_1_	*T*_2_	*T*_3_	*T*_4_	*T*_5_
*a*/*R*	*S*_1_	*S*_2_	*S*_3_	*S*_4_	*S*_5_
0.5	1.387239	0.594892	0.421949	0.428353	0.347941
	1.257488	0.563966	0.406869	0.454861	0.336447

**Table 3.** The first five angular constants of $A_{i}(\beta )$ and $B_{i}(\beta )$.

*A*_1_	$1-4\;\sin^{2}\beta$
*A*_2_	$8\sin^{2}\beta -32\sin^{2}\beta \cos^{2}\beta$
*A*_3_	$-12\sin^{2}\beta +144\sin^{2}\beta \cos^{2}\beta -192\sin^{2}\beta \cos^{4}\beta$
*A*_4_	$16\sin^{2}\beta -384\sin^{2}\beta \cos^{2}\beta +1280\sin^{2}\beta \cos^{4}\beta -1024\sin^{2}\beta \cos^{6}\beta$
*A*_5_	$-20\sin^{2}\beta +800\sin^{2}\beta \cos^{2}\beta -4800\sin^{2}\beta \cos^{4}\beta +8960\sin^{2}\beta \cos^{6}\beta -5120\sin^{2}\beta \cos^{8}\beta$
*B*_1_	1
*B*_2_	$-5+8\cos^{2}\beta$
*B*_3_	$-3+8(1-2\cos^{2}\beta )(2-3\cos^{2}\beta )$
*B*_4_	$3+16(1-2\cos^{2}\beta )-12{\left(1-2\cos^{2}\beta \right)}^{2}-32{\left(1-2\cos^{2}\beta \right)}^{3}$
*B*_5_	$5-16(1-2\cos^{2}\beta )-60{\left(1-2\cos^{2}\beta \right)}^{2}+32{\left(1-2\cos^{2}\beta \right)}^{3}+80{\left(1-2\cos^{2}\beta \right)}^{4}$

The crack makes an angle *β* with respect to the loading direction. By changing *β*, different combinations of mode I and mode II are achieved. When *β* is zero, the specimens are loaded in pure mode I and the mode II stress intensity factor is zero ($K_{II}=0$). For specific inclination angles *β*, the mode I stress intensity factor is zero ($K_{I}=0$) and the specimen is subjected to pure shear load. The angle corresponding to pure shear mode depends on the *a/R* and falls roughly between 22° and 23° [31].

### 1.2. Experimental procedures

To study the influence of corrosive environment on the mechanical behaviour of mixed fracture toughness, the specimens were immersed in half-saturated brine (18 g ml^−1^ at 20°C; 18.5 g ml^−1^ at 50°C; 19.2 g ml^−1^ at 80°C) and at three different temperatures; the temperatures were 20°C, 50°C and 80°C. Thus, there were three different sets of experimental conditions. For comparison, we also conducted tests in the dry state at room temperatures. Four specimens were tested under each set of conditions; thus, 96 tests were performed in total. The soaking time was as long as one month.

Experimental process: (i) Put 10 000 ml of the pre-prepared liquid (pure water, half-saturated brine, or saturated brine) into dry and clean reagent bottles and mark them with numbers. (ii) Heat each bottle using the self-heated system with constant temperature (SHSWCT) for approximately 20 min to ensure that the liquid temperature in each bottle rises to the predetermined temperature. (iii) Gently place each specimen into the correct bottle filled with liquid and seal each bottle to ensure that the concentration of the solution in the bottles does not change via heating. Maintain a stable temperature for the predetermined duration. (iv) Tweeze the specimens out of the bottles lightly to make sure that the specimens are not damaged by the tweezers, and dry the surface of each specimen with filter paper. (v) Dry the specimens using a drying oven for approximately 10 min at 30°C to ensure that the specimens are desiccated. (vi) Test the fracture load of the treated specimens.

## 2. The results and discussion

**Figure 4.**
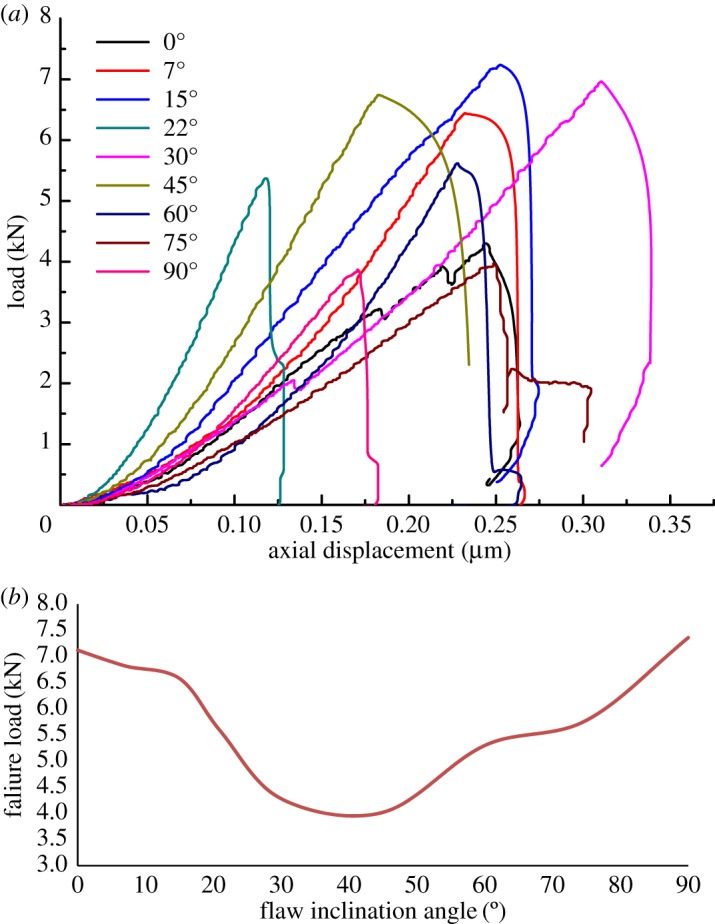
(*a*) Typical failure curves of the dry CSNBD specimens under mixed mode loading (*β* = 0°, 7°, 15°, 22°, 30°, 45°, 60°, 75°, 90°). (*b*) Flaw inclination angle versus failure load.

Typical load-displacement curves for the dry specimens with varying flaw inclination angles *β*, are illustrated in figure 4*a*. Initial crack propagation in the tip of pre-existing flaw occurred in a steady manner during which the load increases. Then, the specimens fail immediately beyond their ultimate load-bearing capacity. Therefore, in this paper, the maximum load can be regarded as the critical fracture load. At the initial stage of loading, the specimens were subjected to a large deformation, which is the same as the results of Almeida's study [32]. The fracture load first decreases then increases with the increase of flaw inclination angle (figure 4*b*). The minimum failure load was observed at a flaw inclination of 45°. Note that the experimental results are totally different from Al-Shayea's [17] results, in which the failure load first increases then decreases. This is because the gypsum interlayers, which can be seen as the typical soft rock, behave in strong plastic deformation when under load. Besides, the width of flaw is just 0.3 mm. This means that the flaw will close when under load due to the good deformation ability, even if the measured stress is less than yield strength. Then, the specimens which have the closed flaw are analogous to Brazilian specimens containing single weak plane. According to the study of Shiyu *et al.* [33] and Xiangchu *et al.* [34], the effect of friction coefficient should be taken in account. Interestingly, a semi-empirical formula which combines with the friction coefficient and crack inclination angle was proposed by the authors as shown below (for more details see Shiyu *et al.* [33] and Xiangchu *et al.* [34]).

2.1 $$P=\frac{2K_{IIC}}{\sin2\beta -f(1-\cos2\beta )\sqrt{\pi a}},$$

where *P* is the failure load and *f* is the friction coefficient between flaw surface.

**Figure 5.**
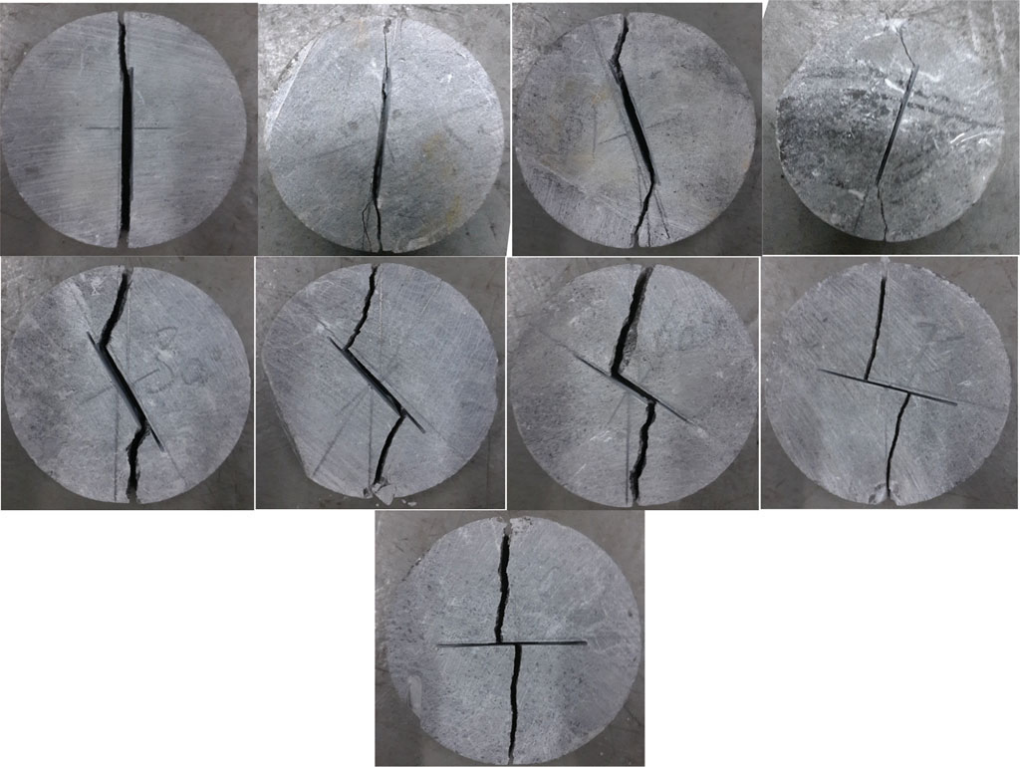
Dry specimens after failure for different inclination angles (*β* = 0°, 7°, 15°, 22°, 30°, 45°, 60°, 75°, 90°).

From equation (2.1), it can been deduced that the failure load first decreases then increases with the increase of *β* when maintaining the *f* constant. The variation trend of equation (1.3) is significantly consistent with our experimental results. However, for *β* = 0° or 90°, the equation fails in predicting the failure load. This is because the flaw surfaces do not close when *β* = 0°. Thus, the friction coefficient is neglected; when *β* = 90°, the specimen failed in a tensile splitting mode rather than a fracture toughness mode. As can be seen from figure 5, the crack did not propagate from the tip of the pre-exiting flaw, but from the centre of flaw, which is similar to fracture morphology of the Brazilian splitting specimen. Therefore, it is interesting to note that the failure load with flaw inclination angle *β* of 90° is larger than 0°. Undoubtedly, equation (2.1) does not apply in the two cases.

### 2.1. The crack propagation trajectories of dry specimens

Figure 5 showed the typical crack propagation trajectories that initiate from the specimens with varying flaw angle of *β* equal to 0°, 7°, 15°, 22°, 30°, 45°, 60°, 75°, 90°. These microscopic fracture curves (i.e. trajectories) consisted of tensile crack and shear crack appeared at *β* equal to 7°, 15°, 22°, 30°, 45°, 60° and 75°, respectively; the new cracks initiated from the flaw notch and propagated along a curved path toward the load point. Yet, the pure tensile cracks only developed in the flaw tip regions of *β* equal to 0° and 90°, and the new cracks were almost orthogonal to the vertical load direction.

**Figure 6.**
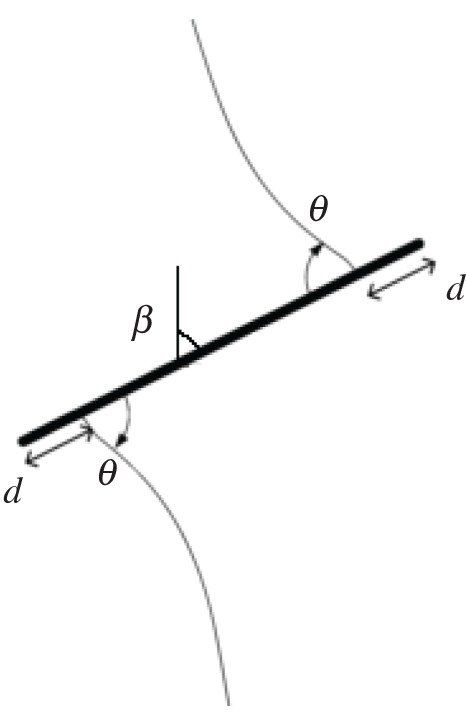
Flaw initiation angle and crack initiation angle.

According to the study of Al-Shayea [17], crack initiation includes two parts: crack initiation angle (θ) and crack initiation location (*d*) (i.e. the location on the flaw periphery relative to the flaw tip) as denoted below (figure 6). From figure 5, one can obtain that the crack initiated at the notch of flaw for values of *β* ranging from 0° to 22°. However, for 22° < *β* < 90°, the location gradually diverted from the tip toward the middle of the pre-existing flaw, and the value of *d* increased as *β* increased. This can be attributed to the fact that the effect of tensile splitting is more evident when *β* is within this range. In other words, the flaw inclination angle at low values (i.e. *β *< 22°) can prevent the development of tensile splitting. Note that the phenomenon is common for all the specimens with the a/r of 0.5. However, a variety of crack initiation angles and crack initiation locations can be obtained by varying the flaw length for a particular inclination angle. For comparison, based on previous studies, the critical angle γ (i.e. the critical angle which the cracks did not initiate from the flaw tip) versus the flaw length are listed in table 4. As can be seen, the critical angle decreases with the increase of flaw length. This implied that the length of flaw can impact the effect of tensile splitting.

**Table 4.** Variation of a/r and γ.

a/r	critical angle (γ)	
0.15	75°	Ghazvinian *et al.* [35]
0.15	75°	Haeri *et al.* [36]
0.30	60°	Ghazvinian *et al.* [35]
0.30	60°	Alshayea [37]
0.45	30°	Ghazvinian *et al.* [35]
0.48	<28°	Erarslan & Williams [38]
0.5	22°	this paper

**Figure 7.**
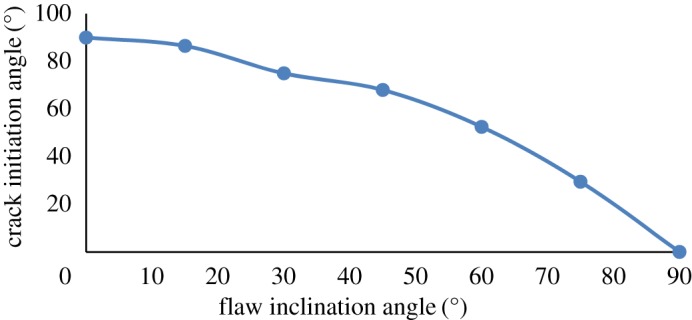
Flaw inclination angle versus crack initiation angle.

As shown in figure 7, the crack initiation angle was 0° for *β* = 0° (i.e. the cracks were parallel to the flaw inclination angle). Then, the crack initiation angle gradually increased as *β* increases. When *β* = 90°, the crack initiation angle reached the maximum value (i.e. 90°).

### 2.2. Effect of water-chloride ion-temperature

**Figure 8.**
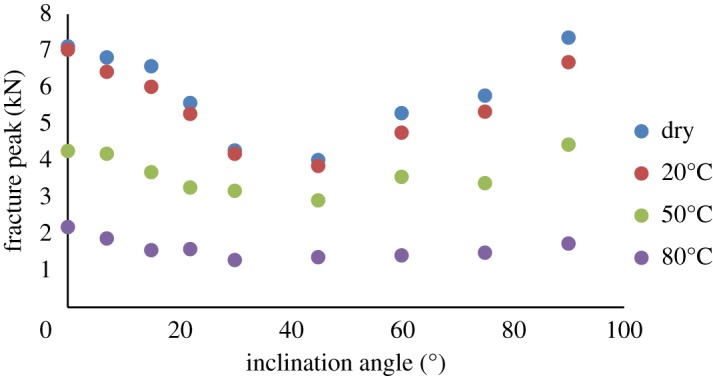
Fracture peak of gypsum specimens under various conditions and pre-crack angle.

**Figure 9.**
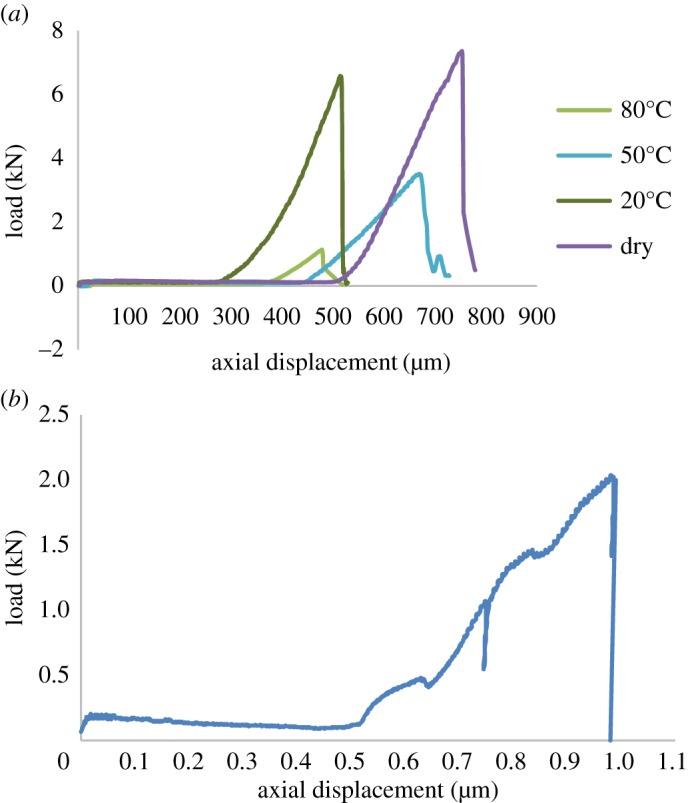
(*a*) Applied load versus axial displacement during the fracture toughness test. (*b*) Load-displacement profile characterized by distinct peaks.

Our results showed that gypsum specimens suffer different degrees of weakening when soaked in different brine (figure 8 and table 5). Brine corrosion resulted in the transformation of the brittle failure of the gypsum into ductile failure. When not soaked in brine, the gypsum specimens exhibited more plastic behaviour, and fracturing was slow. By contrast, the specimens in the dry state exhibited brittle behaviour, and the fracturing was rapid. Under the same soaking conditions, some variations in fracture toughness were observed because the material was heterogeneous and the chemical composition and grain sizes were not exactly uniform for all specimens. Furthermore, because of the different thermo-hydro-chemical interactions, the weakening degree of gypsum also differed. The typical stress–strain curves are illustrated in figure 9*a*. It can be seen that the figure shows curves with various slopes and different fracture load; the dependency of fracture load on brine temperature was evident from the curves; higher brine temperatures were associated with larger deformation. The sudden drop of fracture load is thought to be the effect of water temperature, which causes the specimens to become soft. This is because water can easily penetrate into the interior of the gypsum via capillary action due to the hydrophilic nature of gypsum. Based on the study of Meng *et al*. [24,39], the water inside the gypsum may be bound or absorbed water, which not only increases its porosity but also causes all grains boundaries to open. Besides, the calcium sulphate dihydrate (CaSO_4_·2H_2_O) crystal belongs to the monoclinic crystal series. This atomic structure allows for the perfect cleavage of gypsum. Because different layers are bound via hydrogen bonds, the bonds can be easily broken by forces such as heat or water. This bond breakage can dislocate the layer structure and separate the chains of molecules, eventually increasing the porosity and decreasing the strength of the material. Note that specimens soaked in brine at 80°C showed a distinct chipping phase which is represented by peaks in the profile (figure 9*b*). The difference was due to narrow micro-cracks in the specimens soaked in brine at 80°C. When under loading, the micro-crack initiation, propagation, coalescence and interaction processes induced the distinct peaks.

**Table 5.** Fracture peak of gypsum specimens under various conditions and pre-crack angles.

inclination angle	failure peak-dry (kN)	failure peak-20°C (kN)	failure peak-50°C (kN)	failure peak-80°C(kN)
0°	7.12	7.03	4.27	2.19
7°	6.82	6.43	4.19	1.88
15°	6.58	6.02	3.69	1.56
22°	5.58	5.28	3.27	1.59
30°	4.28	4.19	3.18	1.29
45°	4.02	3.86	2.92	1.37
60°	5.30	4.77	3.56	1.42
75°	5.78	5.34	3.39	1.49
90°	7.36	6.69	4.44	1.74

**Figure 10.**
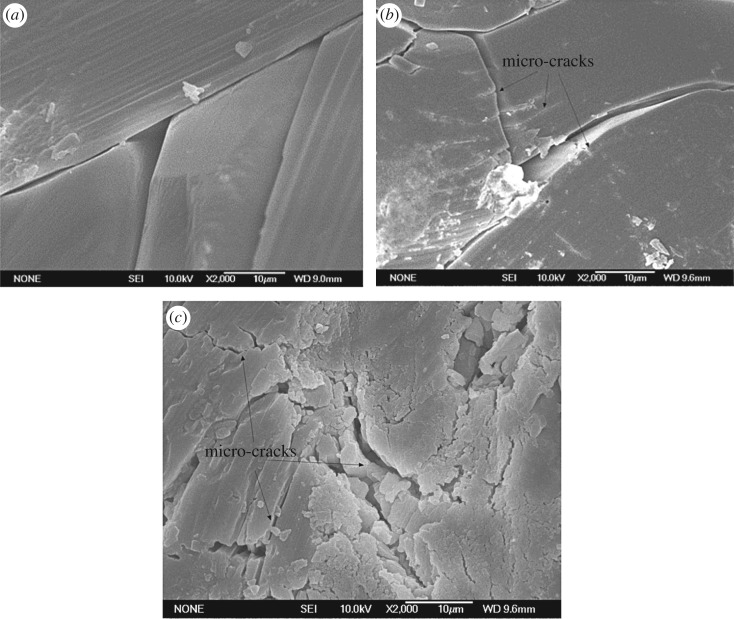
SEM morphologies of gypsum grain boundaries and micro-cracks (magnified 2000×). (*a*) Specimens soaked in brine at 20°C for 24 h. (*b*) Specimens soaked in brine at 50°C for 24 h. (*c*) Specimens soaked in brine at 80°C for 24 h [39].

**Figure 11.**
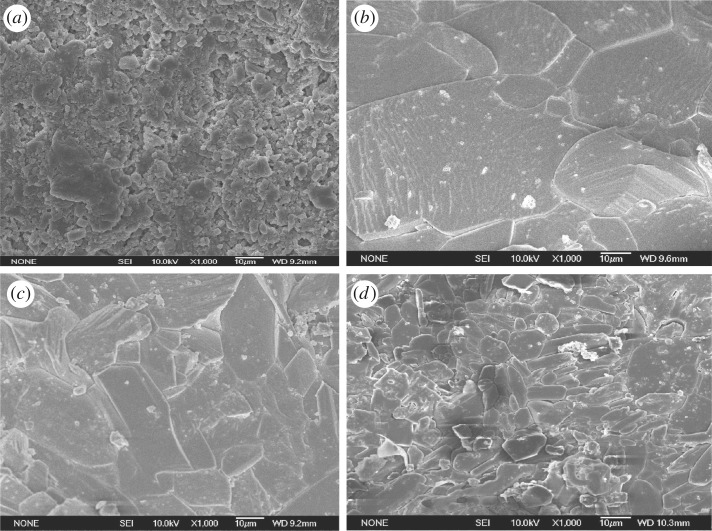
SEM morphologies of gypsum crystal cluster (magnified 1000×). (*a*) Dry specimen. (*b*) Specimens soaked in brine at 20°C for 24 h. (*c*) Specimens soaked in brine at 50°C for 24 h. (*d*) Specimens soaked in brine at 80°C for 24 h [23].

Figures 10 and 11 show the evolution of grain boundary and micro-cracks as a function of brine temperature for specimens measured in SEM. The images which were used to infer the micro-crack density were taken from the surface of treated specimens. As seen in the images (figure 10), the surface of dry specimens exhibited a honeycomb structure. However, when soaking in water, the honeycomb disappeared, and the cement adhering to the crystal was dissolved. Accordingly, the crystal boundaries separated and became clearer. By comparing the morphologies of the gypsum that was soaked in liquid at different temperatures, a significant deterioration could be observed. This deterioration appeared to manifest itself in different ways: the boundaries appeared to become more open between the crystals with increasing brine temperature, and tensile cracks appeared on the gypsum crystals when soaked in 50°C brine (figure 10*b*). When the specimens were soaked at 80°C, the intensity of deterioration became more severe due to the presence of more numerous cracks as shown in figure 10*c*. Obviously, the qualitative observation on micro-crack morphologies are in close agreement with the experimental results of fracture toughness; there is a negative correlation between fracture toughness and micro-crack density. These agreements are excellent, and thus give a strong support to our explanation mentioned above. Besides, the good correlation between the crack density and the values of fracture toughness illustrated the crucial role played by the coupled brine solution and temperature.

**Figure 12.**
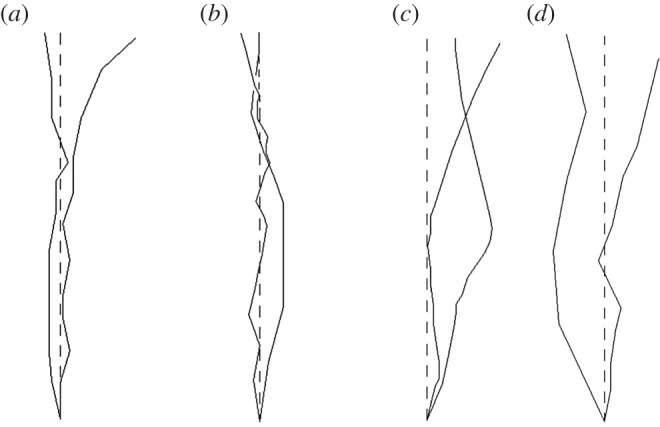
Fractures trajectories observed on the specimen surfaces of gypsum at various conditions. (*a*) Dry specimens. (*b*) Specimens soaked in brine at 20°C. (*c*) Specimens soaked in brine at 50°C. (*d*) Specimens soaked in brine at 80°C.

Kataoka *et al.* [19] asserted that the fracture trajectory (or fracture geometry) observed near the flaw tip is closely related to the fracture initiation of rock and the surrounding environments. Therefore, the analysis of fracture trace is necessary. In this study, we only analysed the traces of Mode I fracture because of the huge data. Figure 12 shows the variation of fracture traces. It can be obtained that all fractures did not propagate in the expected direction. Besides, the deviation of fracture was greater with the increase of brine temperature. This may be because the gypsum grains can be broken into discontinuous and small crystal clusters (i.e. fragmentation) owing to the damage effect of brine solution and heat [23]. The higher the brine temperature was, the larger was the crowding level of crystal cluster. Subsequently, the fractures will tend to avoid going across grains and propagate along grain boundaries, which caused the fracture geometry to be more tortuous. Undoubtedly, the resistances of fractures propagating along boundaries is smaller than that going across grains, and less energy is needed. This can strongly support the dependence of fracture toughness on brine temperature.

### 2.3. Comparison with fracture criteria

Previously, some researchers concentrated on the application of fracture criteria. For example, The maximum tangential stress criterion, the minimum strain energy density criterion, the G criterion and the Gomez criterion have be used to predict the direction of fracture [40–43]. It can be considered that these conventional fracture criteria cannot provide good agreement with the experimental results. In other words, no special fracture criteria can be applied to all cases. For example, the experimental data showed that $K_{IC}$ is less than $K_{IIC}$, while these fracture criteria showed the opposite. Besides, the appropriate fracture criterion should be selected based on the rock type or rock mass properties. Therefore, a modified maximum tangential stress (MMTS) criterion which was proposed by Smith *et al*. [44] is illustrated in this section for predicting the outcomes of experiments. The advantage of the MMTS criterion is that it adopts a more accurate formula for the tangential stress around the crack tip by taking into account *T* stress and fracture process zone (FPZ) (or $r_{c}$).

In the following section, we compare the predictions of the conventional criteria mentioned above with the experimental results (i.e. *β *= 0°, 7°, 15°, 22°); in addition, the application of MMTS associated with the FPZ size is presented.

#### 2.3.1. Mixed mode I-II

**Figure 13.**
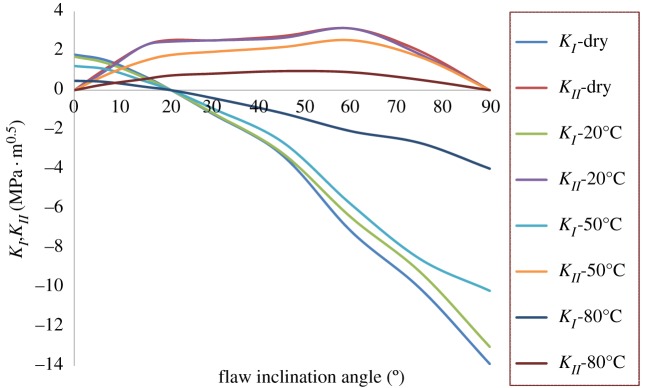
Comparison of mixed mode (I-II) fracture toughness of gypsum under corrosive environment.

**Figure 14.**
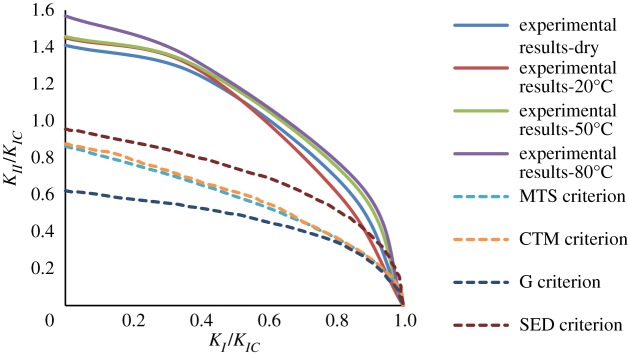
Fracture envelopes for gypsum specimens under corrosive environment and the conventional criteria [40–43].

By using equations (1.1) to (1.4), the mixed mode (I-II) fracture toughness of gypsum under corrosive environment is calculated. As shown in figure 13, when *β* becomes larger, $K_{I}$ decreases and $K_{II}$ increases and hence the fracture pattern of the specimen shifts from pure mode I to pure mode II. Taking specimens soaked in brine at 80°C and dry specimens as examples, pure mode I of specimens soaked in brine at 80°C was $0.477\;\text{MPa}\;{\text{m}}^{0.5}$ with a 379% decrease from that at dry state, while pure mode II ($K_{II}$) was $0.74\;\text{MPa}\;{\text{m}}^{0.5}$ with a 338% decrease from that at dry state. As a result, it was found that the ratio of $K_{IIC}$/$K_{IC}$ (specimens soaked in brine at 80°C) was 1.56, whereas the dry specimens had a value of 1.41. Subsequently, the normalized fracture toughness values of ($K_{I}/K_{IC}$) and ($K_{II}/K_{IIC}$) were determined for specimens under corrosive environment. The plot of ($K_{II}/K_{IIC}$) versus ($K_{I}/K_{IC}$) is referred to as the fracture toughness envelope, which is the fracture locus for the general mixed mode I–II loading. When a point ($K_{I}/K_{IC}$,$K_{II}/K_{IIC}$) falls on the envelope, crack initiates. Figure 14 shows the comparison between the conventional fracture criteria and experimental fracture envelopes. As can be seen, the experimental envelopes derive from the conventional fracture criteria and lie outside these criteria. Besides, the experimental envelopes are dependent on the brine temperature.

#### 2.3.2. The application of modified maximum tangential stress

Ayatollahi and his team members have successfully predicted the onset of fracture of some common materials using MMTS. It was found that MMTS showed a significant agreement with the data tested (e.g. rock, glass, graphite, ceramic, concrete, PMMA) [45,46]. In this section, we employ the criterion to compare with the experimental envelopes at different conditions. The equations with respect to MMTS are listed below:

2.2 $$\begin{array}{r} {}[K_{I}\sin\theta_{0}+K_{II}(3\cos\theta_{0}-1)]-\frac{16T}{3}\sqrt{2\pi r_{c}}\cos\theta_{0}\sin\frac{\theta_{0}}{2}=0, \end{array}$$

2.3 $$\begin{array}{r} \frac{K_{IC}}{K_{I}}=\cos\frac{\theta_{0}}{2}\left[\cos^{2}\frac{\theta_{0}}{2}-\frac{3}{2}\frac{K_{II}\sin\theta_{0}}{K_{I}}\right]+B\sqrt{\frac{2r_{c}}{a}\left(1+\frac{K_{II}^{2}}{K_{I}^{2}}\right)}\sin^{2}\theta_{0}, \end{array}$$

2.4 $$\begin{array}{r} \frac{K_{IC}}{K_{II}}=\cos\frac{\theta_{0}}{2}\left[\frac{K_{I}}{K_{II}}\cos^{2}\frac{\theta_{0}}{2}-\frac{3}{2}\sin\theta_{0}\right]+B\sqrt{\frac{2r_{c}}{a}\left(1+\frac{K_{I}^{2}}{K_{II}^{2}}\right)}\sin^{2}\theta_{0}, \end{array}$$

2.5 $$\begin{array}{r} B=\frac{T\sqrt{\pi a}}{\sqrt{K_{I}^{2}+K_{II}^{2}}} \end{array}$$

2.6 $$\begin{array}{r} \;\text{and}\;T=\frac{T^{*}P}{\pi Rt(R-a)}, \end{array}$$

where $\theta_{0}$ is the angle of maximum tangential stress and *T* is the stress.

The steps used to theoretically predict the onset of fracture are shown below:

(1) By using the values of a/R and *β* reported for gypsum specimen, the crack tip parameters (i.e. $K_{I},K_{II},T$) are determined through finite-element calculation.
(2) Calculate the angle of maximum tangential stress $\theta_{0}$ using equation (2.2).
(3) Calculate the numerical values of $K_{I}/K_{IC}$ and $K_{II}/K_{IC}$ using equations (2.3), (2.4), (2.5) and (2.6).
(4) Determine a point corresponding to each pair of $K_{I}/K_{IC}$ and $K_{II}/K_{IC}$ in a diagram of ($K_{I}/K_{IC}$)/($\;K_{II}/K_{IC}$).
(5) Repeat steps (1)–(4), and determine similar points for another mixed mode.

In addition, several points require attention, as shown below:

(1) The critical distance $r_{c}$ in rocks is equal to the FPZ size in front of the crack tip. This distance is generally assumed to be the inherent property of a material, and is independent of geometrical dimensions of specimens, loading conditions and mode mixture [47].
(2) Some researchers have proposed an equation that describes the FPZ size, as shown in equation (2.7) [48,49].
  2.7 $$r_{c}=\frac{1}{2\pi }{\left(\frac{K_{IC}}{\sigma_{t}}\right)}^{2},$$
  where $\sigma_{t}$ is the tensile strength of the rock. The ratio of $K_{IC}$ to $\sigma_{t}$ is a constant value for gypsum and is independent of the environment [50,51]. Thus, from equation (2.7), $r_{c}$ is a constant value. However, Akbardoost *et al*. and Ayatollahi *et al*. argued that the value of $r_{c}$ is closely related to the MMTS prediction [45,46]. They found that $r_{c}$ is dependent on the size of the specimen, that is, when an appropriate value of $r_{c}$ is used, the MMTS criterion is strongly consistent with the experimental results. Besides, the value of $r_{c}$ increases with increasing specimen size, implying that $r_{c}$ should not be a constant value, which contradicts equation (2.7).
(3) Based on equations (2.3) and (2.4), the values of $K_{I}/K_{IC}$ and $K_{II}/K_{IC}$ change with varying $r_{c}$, leading to different fracture envelopes. Figure 14 shows that the experimental fracture envelopes are scaled down with increasing temperature and that $r_{c}$ is dependent on the solution temperature.

**Figure 15.**
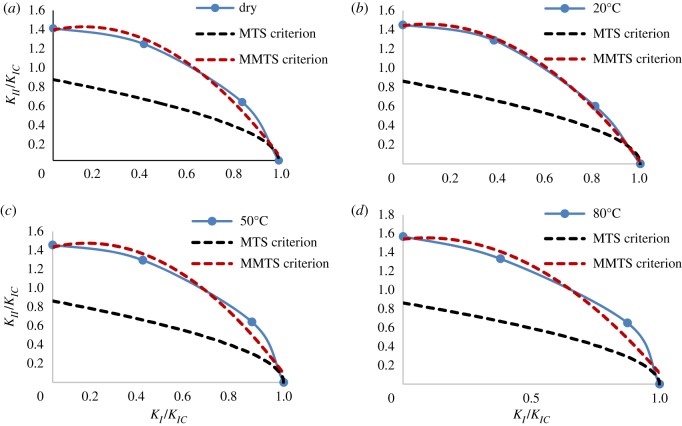
Test data obtained from fracture tests on gypsum using the CSNBD specimens in comparison with theoretical predictions of commonly used fracture criteria. (*a*) *r*_c_ = 2.13 mm. (*b*) *r*_c_ = 2.24 mm. (*c*) *r*_c_ = 2.53 mm. (*d*) *r*_c_ = 3.02 mm

Based on previous studies, this paper assumes that the MMTS criterion is the optimum model for fitting the experimental data. Equations (2.2) to (2.6) were then input into Matlab to invert the parameter (i.e. FPZ) using a back-propagation neural network [52]. Then, the FPZ values under different conditions can be obtained. As shown in figure 15, fair predictions were obtained for the mixed mode fracture resistance of gypsum under varying brine temperatures; in addition, the FPZ value increases with increasing brine temperature. The more severe weakening of gypsum, the larger was the FPZ size.

## 3. Conclusion

The mixed fracture toughness values, fracture traces and FPZ of gypsum interlayers soaked in brine with various temperature were investigated in the laboratory. In this study, the following main conclusions were obtained.

When at a given inclination angle, the fracture toughness of gypsum soaked in brine decreased with increasing brine temperature. When at a given brine temperature, the fracture load of gypsum first decreased then increased with the increasing of inclination angle.

When soaked in brine at 20°C, the crystal boundaries of gypsum appeared open between the crystals. When soaked in 50°C brine, a small number of tensile cracks which were perpendicular to the crystal boundaries began to appear on the gypsum crystals. However, when the specimens were soaked at 80°C, the intensity of deterioration became the most severe due to the presence of more numerous cracks.

The experimental fracture envelope derived from the conventional fracture criteria and lay outside these criteria. The experimental envelopes were gradually scaled up with the increase of brine temperature, which are greatly dependent on brine temperature. The size of FPZ was greatly dependent on the damage degree of materials and gradually increased with increase of brine temperature.

## Data accessibility

The datasets supporting this article have been uploaded to Dryad: https://doi.org/10.5061/dryad.sg2d3 [54].
